# Indocyanine Green (ICG) Fluorescence Is Dependent on Monomer with Planar and Twisted Structures and Inhibited by H-Aggregation

**DOI:** 10.3390/ijms241713030

**Published:** 2023-08-22

**Authors:** Bonghwan Chon, William Ghann, Jamal Uddin, Bahman Anvari, Vikas Kundra

**Affiliations:** 1 Department of Diagnostic Radiology and Nuclear Medicine, University of Maryland School of Medicine, 22 S. Greene St., Baltimore, MD 21201, USA; bonghwan.chon@som.umaryland.edu; 2 Center for Nanotechnology, Department of Natural Sciences, Coppin State University, 2500 W North Ave, Baltimore, MD 21216, USA; 3 Department of Biochemistry, University of California, Riverside, 900 University Ave, Riverside, CA 92521, USA; 4 Department of Bioengineering, University of California, Riverside, 900 University Ave, Riverside, CA 92521, USA; 5 Marlene and Stewart Greenebaum NCI Comprehensive Cancer Center Program in Oncology, Experimental Therapeutics, University of Maryland School of Medicine, 22 South Greene Street, Baltimore, MD 21201, USA

**Keywords:** ICG (indocyanine green), near-infrared (NIR), fluorescence, twisted intramolecular charge transfer (TICT)

## Abstract

The optical properties of indocyanine green (ICG) as a near-infrared (NIR) fluorescence dye depend on the nature of the solvent medium and the dye concentration. In the ICG absorption spectra of water, at high concentrations, there were absorption maxima at 700 nm, implying H-aggregates. With ICG dilution, the main absorption peak was at 780 nm, implying monomers. However, in ethanol, the absorption maximum was 780 nm, and the shapes of the absorption spectra were identical regardless of the ICG concentration, indicating that ICG in ethanol exists only as a monomer without H-aggregates. We found that emission was due to the monomer form and decreased with H-aggregate formation. In the fluorescence spectra, the 820 nm emission band was dominant at low concentrations, whereas at high concentrations, we found that the emission peaks were converted to 880 nm, suggesting a new form via the twisted intramolecular charge transfer (TICT) process of ICG. The NIR fluorescence intensity of ICG in ethanol was approximately 12- and 9-times brighter than in water in the NIR-I and -II regions, respectively. We propose an energy diagram of ICG to describe absorptive and emissive transitions through the ICG structures such as the monomer, H-aggregated, and TICT monomer forms.

## 1. Introduction

Indocyanine green (ICG) is a near-infrared (NIR) fluorescent dye that is widely used as an image contrast agent for medical diagnosis [1]. It was developed in the Second World War as a dye for use in photography. It was first approved for clinical use in humans in 1956 and tested in 1957 for use in human medicine [2]. In 1959, it became the only NIR dye approved by the FDA (US Food and Drug Administration) for clinical applications such as angiography, lymphatic, biliary, and intestinal functional imaging, dental imaging, and oncologic image-guided surgery [3,4,5,6,7,8,9]. In addition, there is increasing interest in broader applications of NIR fluorescence imaging in the NIR-I (700–900 nm) and -II (1000–1700 nm) (the first and second windows of NIR) with ICG and ICG formulations such as liposomes and micelles [10,11]. Recently, it was reported that ICG has a long-wavelength emission tail that could extend into NIR-II, also referred to as the short-wave infrared (SWIR) region [11,12]. Optical imaging using the NIR-II region would improve resolution with high imaging contrast due to low background, and increased penetration depth of the longer wavelengths through tissue compared with optical imaging in the NIR-I or visible region.

**Figure 1:**
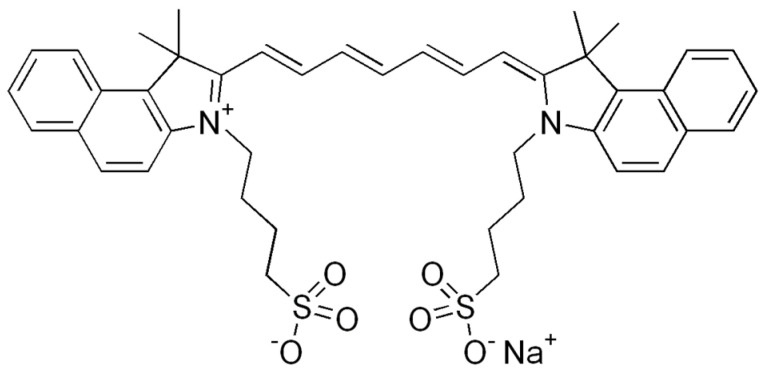
Chemical structure of indocyanine green (ICG).

ICG is a water-soluble anionic tricarbocyanine dye containing an extended seven-carbon p-conjugated system. The chemical structure of ICG is shown in Figure 1. Its chemical structure consists of polycyclic moieties connected by a long carbon chain. While the polycyclic moieties are responsible for the lipophilic properties of ICG, the sulfate groups impart hydrophilicity [13]. As a result, the amphiphilic property of ICG is organic-soluble, such as in ethanol and DMSO, as well as water-soluble, including in various buffer solutions. ICG has a poor solubility of up to ~1 mg/mL and a low fluorescence quantum yield in an aqueous solution [13,14]. In terms of structural stability, the cyanine backbone’s basic structures can result in aggregation. At higher concentrations, dimers or aggregates having different spectral properties are formed, including poorly emissive forms. Self-assembled ICG depends on the concentration, ionic strength, and polarity of the solvent [15]. Among these factors, H-like aggregation is favorable as the polarity of the media increases [16]. For example, water is a relatively high-polar medium, whereas ethanol is a low polar medium. Theoretically, cyanine dyes may exist in planar or twist forms swiveling at the backbone [17]. The rotation along the π-conjugated backbone of cyanine dye has the potential to red-shift fluorescence [17]. Though theorized, such a twist structure as a dominant form has not been demonstrated experimentally for ICG. Most previous photophysical measurements have been studied on visible detectors such as silicon-based cameras or photomultiplier tubes, limiting our understanding of the absorptive and emissive properties of ICG in different solvents in the >800 nm NIR I and NIR II range [12,13]. Therefore, the goals of this study were to evaluate the photophysical properties of ICG in two solvents (water and ethanol) with different polarities through absorption and fluorescence spectroscopy at broader NIR-I and -II wavelengths to identify the absorbed and emitted energy states of ICG molecular structures.

Here, we report the unique optical properties, such as absorption and fluorescence, as a function of ICG concentration. These fluorescence spectra were detected with an indium gallium arsenide (InGaAs) sensor for the NIR emission properties, ensuring the collection of the full emission spectra of ICG. Based on the absorption and fluorescence spectra of ICG, we found different concentration-dependent molecular structures, such as H-aggregated, and two different monomer structures, normal vs. twisted forms. In a concentration-dependent fluorescence spectrum, our findings suggest that the ICG dye emission wavelength enables the second window of NIR imaging. We aim for our findings to be useful in understanding the photophysical properties of ICG in changing solvent environments and the aggregation/emission effects as a function of ICG concentration, enabling further applications.

## 2. Results

### 2.1. Absorption Spectra of ICG in Water and Ethanol

The solvents we chose to evaluate the photophysical properties were water as an aqueous medium and ethanol as an organic solvent reference since ICG has amphiphilic solubility. Figure S1a–d show the absorption spectra of ICG at different concentrations dissolved in water and ethanol. We recorded the absorption spectra in two different pathlength cells for enhanced dynamic range of absorbance by 2.5 mm for the low concentration and 0.5 mm for the high concentration (62.5 μM–500 μM). Absorbance spectra in high concentrations were evaluated by a 5-times pathlength correction factor based on Beer’s Law [18]. By Beer–Lambert Law, the apparent absorbance is proportional to the ICG concentration. 

**Figure 2:**
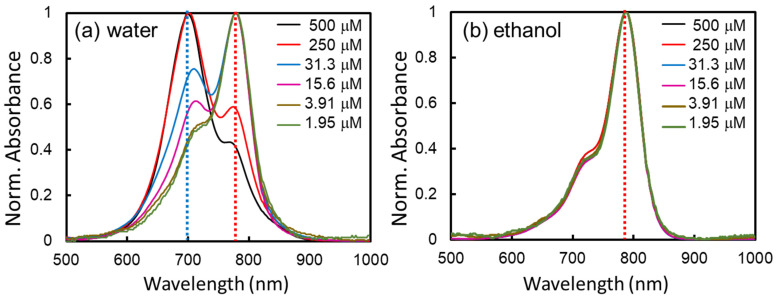
Comparison of the normalized absorption spectra of ICG dissolved in (**a**) water and (**b**) ethanol. All spectra are normalized at the maximum intensity to show the change in spectral shapes. The ICG concentrations are 500, 250, 31.3, 15.6, 3.91, and 1.95 μM, respectively. The guideline is marked by red (780 nm) and blue (700 nm) lines. The absorbance spectra of ICG at 500 μM and 250 μM are pathlength-corrected, as detailed in the experimental section. Unnormalized absorption spectra of ICG in water and ethanol are shown in Supporting Information Figure S1.

To analyze the spectral shape changes and peak shifts due to the ICG concentration, the absorption spectra were normalized by the peak maximum intensity and displayed in Figure 2a,b, in water and ethanol, respectively. In Figure 2a, showing the absorption spectra of ICG in water-solvent, we saw two absorption peaks at around 780 nm and 700 nm, originating from the monomer and H-type aggregated forms, respectively [1,19]. In the absorption spectra shown in Supporting Information Figure S1, we did not see any significant peak around 890 nm, indicating the lack of J-aggregates of ICG [1]. At high concentrations in water, the main peak was at 700 nm, with a shoulder peak at 780 nm. However, with dilution down to 3.9 μM, the peak at 780 nm increased while the 700 nm maximum peak changed into a shoulder. As the ICG concentration increased, the main and shoulder peaks switched in the absorption spectra, indicating the transformation of the ICG monomer to H-type aggregated forms. Contrary to this behavior, the normalized absorption spectra of ICG in ethanol and spectral shapes were identical regardless of the ICG concentration. The peaks of maximum and shoulder were at around 780 nm and 700 nm, respectively. Therefore, unlike in aqueous media, the ICG monomer in ethanol is the dominant form, and the H-type aggregated form is nearly avoided in ethanol.

### 2.2. 2D COS Analysis of ICG Absorption Spectra in Water and Ethanol

**Figure 3:**
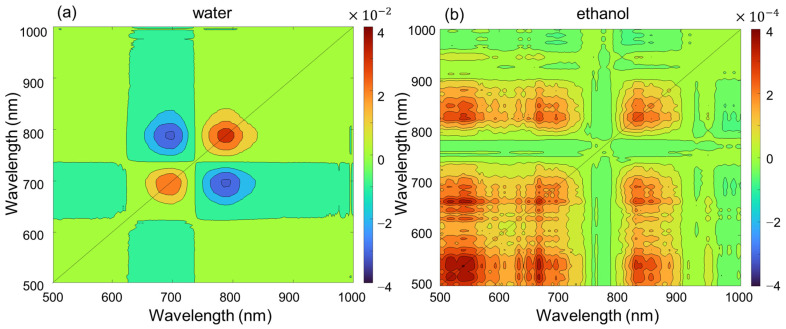
Synchronous 2D correlation spectra obtained from the ICG concentration-dependent absorption spectra in (**a**) water and (**b**) ethanol.

We performed a 2D correlation analysis on the absorption spectra. Here, we monitored the absorption spectra by changing the concentration as a perturbation. Figure 3a,b shows the synchronous 2D correlation spectra in water and ethanol, respectively. The power spectrum along the diagonal line in the synchronous spectrum showed two peaks at 700 nm and 780 nm. The negative cross peak at 780 nm and 700 nm in synchronous correlation spectra suggests that the band at 780 nm decreases while the band at 700 nm increases at the same time. On the other hand, the synchronous 2D correlation spectrum of ICG in ethanol shown in Figure 3b showed no correlation between the 780 nm and 700 nm bands. The correlation values in ethanol were close to zero, approximately 100 times lower than those in water. In addition, there were no negative cross peaks, so the intensity changes of the two bands at 780 nm and 700 nm did not correlate when the ICG concentration changed in ethanol.

### 2.3. NIR Fluorescence Spectra of ICG in Water and Ethanol

**Figure 4:**
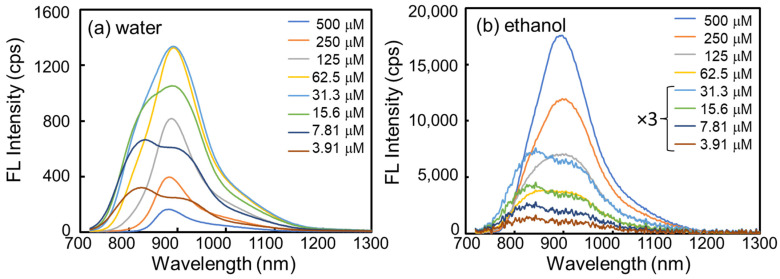
Fluorescence spectra of concentration-dependent ICG in (**a**) water and (**b**) ethanol, respectively. Fluorescence spectra in ethanol at 31.3, 15.6, 7.81, and 3.91 μM are multiplied by a factor of 3 to enhance visibility. Samples were in a microfluorometer cell with a 2 mm path length and were excited at 730 nm.

The visible and NIR optical properties of fluorescent materials are commonly characterized by a spectrometer with a conventional light detector with photomultiplier tubes (PMT) and silicon-based detectors (photodiodes and charge-coupled devices). However, at longer wavelengths, the sensitivity of these detectors suddenly decreases and has extremely low efficiency, so that NIR detection over 900 nm is challenging and one is unable to record the full spectrum. Furthermore, the fluorescence intensity is proportional to the concentration under low concentration conditions, in which the optical density is less than 0.07 [20]. However, high concentrations can decrease the fluorescence intensity and further induce changes in the spectral shape because the fluorescence at shorter wavelengths is absorbed by other molecules of the same species [20]. This can result in variability in emission spectra, as has been reported in the literature [21]. Here, we used a fluorometer cell with a 2 mm thickness and an NIR fluorometer with an InGaAs detector to minimize spectral artifacts without further intensity calibration because detection efficiency is essentially stable at the various wavelengths tested. Figure 4a,b shows the fluorescence spectra of ICG in water and ethanol, respectively. 

In the literature [22,23], it is common to excite ICG at 730 nm to decrease absorption by the same species. We excited at 730 nm, and emission wavelengths were evaluated up to 1400 nm. The dilution series of ICG concentration is the same as in the absorption measurement in Figure 3. Figure 4a presents the fluorescence spectra of ICG in water. With a low concentration of ICG (3.91 μM), the main emission peak was observed at 820 nm, consistent with previous reports [12]. As the ICG concentration increased, the emission band at 820 nm decreased, and a new band around 880 nm was generated. At 62.5 μM ICG in water, the 880 nm band was dominant without showing the 820 nm band. As the ICG concentration increased up to 500 μM, only the 880 nm band was detected, and overall emission intensity decreased. On the other hand, in Figure 4b of the fluorescence spectra in ethanol, the peak was broader at 820 nm and 880 nm at low concentrations, and the 880 nm peak was dominant with increasing concentration. At high concentrations in ethanol (500 μM), only the 880 nm peak was dominant.

**Figure 5:**
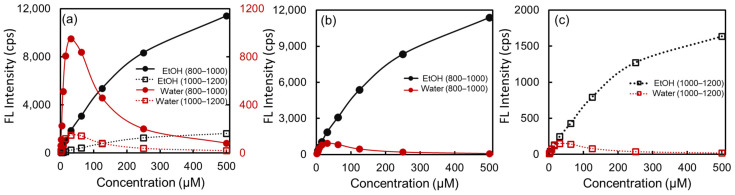
(**a**) Spectrally integrated NIR fluorescent intensity of ICG in DI water (red) and ethanol (black). The closed circles are the emission intensity integrated from 800 nm to 1000 nm as a NIR-I window, whereas the open squares are the emission intensity from 1000 nm to 1200 nm for the NIR-II window. To compare the ICG fluorescent intensity in the two solvents, intensity plots are presented as a function of ICG concentration at (**b**) NIR-I and (**c**) NIR-II using the same fluorescence intensity scale (*y*-axis).

Figure 5 shows the integrated fluorescent intensity as a function of ICG concentration in water (red) and ethanol (black). All spectrally integrated intensity was averaged in two integrated regions: 800 nm–1000 nm (solid circle) and 1000 nm–1200 nm (open square) as the first and second windows of NIR, respectively. Interestingly, the integrated intensity in water increased and then decreased with the ICG concentration. The intensity was maximal at approximately 30–60 μM of ICG in water, with a concentration-dependent emission intensity. However, in ethanol, it kept increasing and began to plateau at the highest concentration experimentally tested, 500 μM. Figure 5b,c presents the fluorescent intensity of ICG in water and ethanol in the NIR-I and -II regions, respectively. At concentrations lower than 30 μM, the intensities of ICG in water and ethanol had a similar increasing behavior as ICG concentration increased, whereas over 30 μM, the concentration-dependent behavior was quite different in the two solvents [14]. The fluorescent intensity of ICG in ethanol kept increasing as the ICG concentration increased, whereas the intensity in water decreased in a concentration-dependent manner [24]. Overall, all concentrations of ICG in ethanol were much brighter than in water. At 30 μM ICG, the emission intensity in ethanol was approximately twice as bright as that in water for both the first and second windows of NIR. At the highest concentration tested, 500 μM of ICG, the fluorescence in ethanol was ~140 or ~85 times brighter than in water at 800–1000 nm or 1000–1200 nm, respectively, when excited by 730 nm light.

### 2.4. Spectral Decomposition of ICG Formations in Absorption Spectra

**Figure 6:**
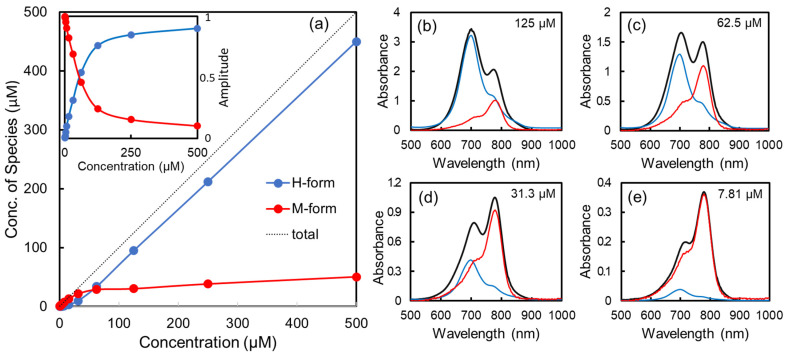
(**a**) Spectral decomposition analysis result of concentration-dependent ICG forms in water. The black line is the total concentration, and the blue (red) lines denote the concentration of H-aggregated (monomer) forms, respectively. Inset is the fitted amplitude from the spectral decomposition. Fitting errors are smaller than their symbols. Representative decomposed plots of a binary mixture (monomer and H-aggregate forms) using absorption spectra of each form: (**b**) 125 μM, (**c**) 65.5 m, (**d**) 31.3 μM, and (**e**) 7.81 μM.

ICG in water exists in two forms: the monomer (M) and the H-type aggregated form (H). For quantitative analyses of M and H-aggregates of ICG in water, the classical least squares method was used to decompose the acquired absorption spectra into a linear combination of two spectra of single-chemical species in M and H forms [1].

(1) $$A_{observed}(\lambda )=f_{M}\times A_{M}(\lambda )+f_{H}\times A_{H}(\lambda )$$

where *A*_M_(λ) and *A*_H_(λ) are the experimentally measured absorption spectra of the pure M- and H-forms as described, respectively; λ is wavelength; and *f*_M_ and *f*_H_ are the predicted mass fractions of M- and H-forms, respectively. Here we assumed that the absorption spectrum in a 1.3 μM water ICG solution would be the same as in pure H-forms because the absorption spectra were not much changed from 250 μM to 500 μM. Moreover, since the spectral shape is not significantly changed below 3.91 μM, we assumed that a 1.56 μM ICG solution would have the absorption spectrum of the pure monomer without any aggregation. Figure 6 inset displays the fitted amplitudes of all concentrations, whereas Figure 6 presents the calculated concentration of M- and H-forms in the given total concentration in water. As the ICG concentration increases in water, the proportion of the monomer increases slightly and then almost plateaus at 50 μM. In 50 μM of ICG solution, the M− and H−forms would be the same at 50%, indicating that 50 μM of ICG solution should be a mixture of 25 μM of monomer and 25 μM of H-aggregated forms. Figure 5b–e show examples of spectral decomposition for a mixture spectrum using the two basis functions of $A_{M}(\lambda )$ and $A_{H}(\lambda )$. The H-forms (blue) are dominant at high concentrations (Figure 6b), whereas the monomer (red) is dominant at low concentrations (Figure 6e). In Figure 6c,d, there is a mixture of the M- and H-forms. On the other hand, ICG in ethanol exists essentially as only the monomer form without H-aggregation at all concentrations tested.

### 2.5. Spectral Analysis of Two Emitting States of ICG Fluorescence Spectra

Broad emission peaks at low concentrations coalesced into two main peaks at higher ICG concentrations. For quantitative analysis of the fluorescence spectra, we performed a global fit of the peak area using two modified Gaussian functions. We settled on two emitting states in the fluorescence spectra to explain the curves. As discussed earlier, the absorption spectrum of the ICG monomer at 1.56 μM was the main band at 780 nm, and a shoulder was seen at 700 nm, indicating the approximately 40% amplitude of the main band. In principle, the absorption and emission spectra have a mirror-image relationship since the absorption and emission processes have the same symmetry due to the same vibrational levels in their ground and excited states [25]. The model for emission spectra has one Gaussian function for the main peak, with 40% of the main peak presenting the redshifted shoulder peak. The centers of the two main bands are 820 nm and 880 nm, originating from the planar and twisted ICG monomer structures, respectively. The rationale for assigning the spectral changes as H aggregation as well as planar and Twist forms of the monomer is further elaborated upon later in the discussion. 

**Figure 7:**
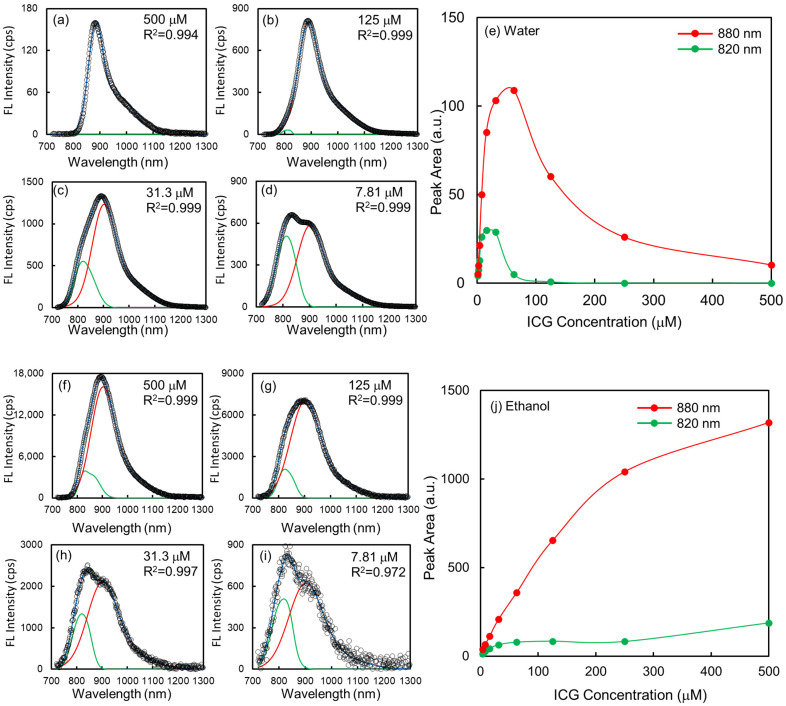
Example of peak fitting of the fluorescence spectra by two basis functions with main and shoulder bands, indicating the mirror image of the absorption spectrum: (**a**,**f**) 500 μM, (**b**,**g**) 125 μM, (**c**,**h**) 31.3 μM, and (**d**,**i**) 7.81 μM, (water and ethanol), respectively. The black circles are the measured fluorescence spectra, and the blue lines are the fitted spectra. The R^2^ values are the regression values between the measured and fitted data. The peak areas of the two basis functions at 820 nm (green, planar monomer) and 880 nm peaks (red, twisted monomer) are shown based on ICG concentrations in (**e**) water and (**j**) ethanol, respectively.

As a model, the following equation was used:

(2) $$F=\sum_{i=1}^{2}a_{i}\cdot exp\left[-{\left(\frac{x-b_{i}}{c_{i}}\right)}^{2}\right]+0.4\times a_{i}\cdot exp\left[-{\left(\frac{x-d_{i}}{e_{i}}\right)}^{2}\right]$$

where *F* is the observed fluorescence spectra, a is the amplitude of the peak, b and d are the center position of the peak, and $c\;and\;e_{i}$ are related to the peak width. The best-fit parameters of the center position (*b* and *d*) are 815 and 890 nm for band 1 and 890 nm and 990 nm for band 2. The difference between the main and shoulder peaks is approximately 1000 cm^−1^. After spectral deconvolution of the fluorescence spectra, the peak centers of the 820 nm and 880 nm bands are shifted to 815 nm and 890 nm, respectively. We found that the observed curve can be satisfactorily fitted by a two-model function. The fitting results are displayed in Figure 7a–d (in water) and f–i (in ethanol) with ICG concentrations at 500 μM, 125 μM, 31.3 μM, and 7.81 μM and regression values of 0.97–0.99. Overall, the model peak areas as a function of ICG concentration are shown in Figure 7e (in water) and Figure 7j (in ethanol), respectively. In water, the peak areas increased and decreased as the ICG concentration increased. The maximum peak area at 820 nm emission is 15.6 μM, whereas the one at the 880 nm band is 62.5 μM. In ethanol, the 820 nm band increases at low concentrations and then plateaus at approximately 62.5 μM, whereas the 880 nm band representing the twisted form keeps increasing as the ICG concentration increases.

## 3. Discussion

In this work, we examined the optical properties of ICG in aqueous and organic solutions employing absorption and fluorescence spectroscopies. We demonstrate that the ICG monomer is the emissive form and the H-aggregates of ICG are non-emissive [25,26]. In addition, the ICG monomer has two emission bands at 820 nm and 880 nm, which originate from the planar or twist ICG formations, respectively. We found the coalescence of ICG’s twisted emission peak at 880 nm experimentally in the fluorescence spectrum for the first time. We note that 12-fold and 9-fold increased fluorescence at low ICG concentrations can be achieved in ethanol versus in water in the NIR-I and NIR-II regions, respectively. In addition, through concentration-dependent absorption spectroscopy, we resolved two absorption peaks at 700 nm and 780 nm in an aqueous solution, originating from H-aggregates and monomers of ICG, respectively. These forms in water have ICG concentration-dependent behavior; the monomer is the dominant form at low concentrations, whereas the H-aggregates are dominant at higher ICG concentrations. We found that two spectral components can be identified in the overall concentration-dependent absorption spectra of water, and the ratio of the two components showed counter-correlated behaviors. The ICG monomer is the only form in ethanol regardless of ICG concentration, and emission in ethanol due to monomer predominance does not peak at low concentrations as does emission in water. 

In the fluorescence spectra of ICG, we can obviously resolve two bands and their dependency on molecular aggregation state as a function of ICG concentration. In addition, modeling supports two dominant peaks that can explain the observed emission spectra, including at NIR-I and NIR-II wavelengths. This finding suggests the possibility of tuning the emissive states of the fluorescent molecule from 820 nm to 880 nm with long tail emission in the second window of the NIR. ICG is a clinically approved NIR-I dye that emits fluorescence in the first window of the NIR. In addition, it has been reported that it may be possible to detect the tail emission from an aqueous solution of ICG on a short-wavelength infrared camera even though the emission peak is centered at 820 nm [11,12,27]. Furthermore, Zhu et al. [17] investigated the NIR-II emission tail of cyanine dyes like ICG. In the ground state, they maintain a mostly planar electron distribution, whereas, after light excitation, the middle carbon-carbon bonds of the conjugated backbone are elongated, providing a smooth rotation in the process of the excited states [17]. As a result, the rotation of the middle p-conjugated backbone structure leads to a charge redistribution that further induces the twisted intramolecular charge transfer (TICT) process. The transition of an environment-sensitive TICT excited state to the ground state results in redshifted emission in the NIR-II region. The simulated results from the density functional theory calculation report ~880 nm TICT emission when the twisted angle achieves around 55° (or 125°) [17]. In addition, the TICT emission intensity compared with the planar geometry is predicted to decrease due to the reduced transition dipole moments between the ground and excited states during the twisting process [17]. We demonstrate for the first time the concentration-dependent existence of the theoretically expected TICT form in the fluorescence spectrum. Here, we report the full emission spectra at the center of the 880 nm peak with a long tail in the NIR-II region. This work suggests that NIR-II fluorophores may be developed by controlling the emission center in the fluorescence spectrum and optimizing the bright NIR-II emission.

**Figure 8:**
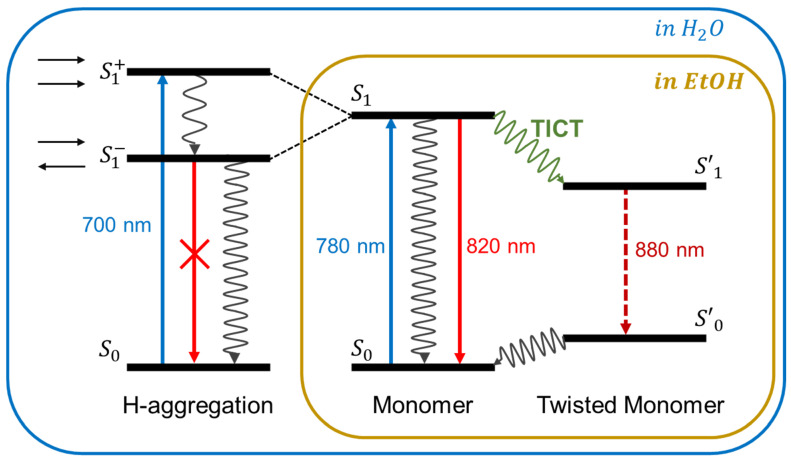
Illustration of the exciton model for the electronic transition of molecular dimers. The ground and the excited states are denoted by $S_{o}$ and $S_{1}$, respectively. The absorption and emission of light between $S_{o}$ and $S_{1}$ are the blue and red arrows, respectively. Transitioning to the twisted internal charge transfer state changes the ground and excited energy states,$S_{0}^{\prime }$ and $S_{1}^{\prime }$, respectively. The H-type aggregation formed by side-by-side association demonstrates strongly increased energy separation between absorbing and emitting states, and, due to their forbidden character, their fluorescence is suppressed. Blue box: forms seen in water. Brown box: forms seen in ethanol.

To account for the optical properties of ICG, we present an energy diagram of exciton in Figure 8 that considers both the absorption and the fluorescence processes, including molecular structural changes such as aggregation and conformational changes. Aggregation is the strong interaction between constituting molecules as a solute and the environment, such as the solvent. Therefore, the exciton is not localized to one monomer, but it may propagate through the H-aggregates. The exciton nature of the optical transition in the aggregation is responsible for optical properties such as the transition energy and oscillator strength. Accordingly, the exciton in the dye can be delocalized across the fluorescent molecules. Based on exciton theory [28], Michael Kasha suggested the first model of electronic coupling with the packing geometry and photophysical properties of molecular aggregates of organic dyes [28]. He studied the effect of the relative orientation of two molecules, which is simplified as their transition dipoles are placed along the long axis of the molecules. For simplicity, the ground-state energy levels are shown as the same for monomers and H-aggregates. The excited states of H-aggregates are split into two levels because of electronic degeneracy. The transition energy and strength depend on the transition dipole moment with a charge distribution of two energy states. When the dipoles have an opposing orientation (S1^−^ in Figure 8), the electronic transition to the lower energy is forbidden and cannot be spectrally observed in the absorption and emission spectra. Instead, the transition to the higher energy state (S1^+^ in Figure 8) is allowed. The electronic transition from the ground state to the excited state will be blue-shifted relative to the monomer in the absorption spectra. In the case of the ICG absorption in water, the peak at 780 nm is shifted to 700 nm at higher concentrations, indicating the generation of H-aggregates. These structures with parallel and anti-parallel dipole moments are characteristic of H-aggregates. The Kasha model also expects the emission properties of H-aggregates, and in general, the radiative decay in H-type aggregates is suppressed because the transition is symmetry-forbidden [28,29]. In water, H-aggregates and monomers were observed, whereas only fluorescing monomers were seen in ethanol (Figure 8, blue vs. brown boxes). The monomer exists in a planar or twist form, resulting in 820 nm and 880 nm emissions (Figure 8).

The optical density at any wavelength depends not only on the nature of the solvent medium but also on the concentration of ICG. We note that the ICG concentration-dependent H-type aggregation in water is consistent with previous reports [1]. This causes the principal peaks in the absorption spectrum to shift from 780 nm to 700 nm with increasing ICG concentration, similar to spectral shifts that have been observed by others [1]. In organic solvents, however, we found that ICG in ethanol avoids the tendency to aggregate, as demonstrated by the spectral shape of the absorption spectra, which essentially remains as a monomer regardless of the ICG concentrations tested [14]. Diluted ICG in water prefers the monomer form, whereas concentrated ICG in water prefers the H-aggregate form. In contrast, ICG in ethanol does not undergo concentration-dependent formational change, so it always exists in the monomer as a conformation even at high concentrations. This result is significant: ~10 times higher emission was found in ethanol than in water at low concentrations, similar to previously calculated emission values based on the product of the extinction coefficient and the fluorescence quantum yield. In the concentration-dependent emission plots (Figure 5), the ratio of the fluorescence intensities in ethanol and water is not constant, indicating the solvent- and concentration-dependent fluorescence processes are complicated. In reports of the fluorescence studies in water and methanol binary solvents, the nonradiative process was suggested to be due to the combination of intersystem crossing, photoisomerization, internal conversion of ICG, and solvent-dependent nonradiative rate [30,31]. Molecular aggregation and TICT processes in different solvents with a dielectric constant are often associated with molecular interaction, solvation, and structural changes within the system [32]. Moreover, the emission bandshape change with increasing concentrations of ICG in water and ethanol is influenced by molecular interactions (solute-solute), solvation interactions (solvent-solute), and the underlying photophysical properties of the system. Molecular aggregation and TICT processes are often associated with molecular interactions and structural changes within the system [33,34]. As the concentration increases, the intermolecular interaction is also altered in a particular solvent, such as due to polarity, which can influence the efficacy of the TICT process [33], consequently influencing the observed emission. Water and ethanol have polarity, water to a greater degree. Concentration-dependent TICT has been reported for cyanoacrylic dye L_1_ by El-Zohry et al. [32], who suggested that in addition to solvent polarity and acceptor strength, H bonding between carboxylic groups with increasing concentration results in dimerization favoring TICT. Molecular alterations such as dimerization with increasing concentration may also be involved in TICT with ICG.

To gain insights into solvent-dependent fluorescence brightness, we compared the integrated intensity of the NIR-I and -II regions at the same ICG concentration in water and ethanol. To quantify the molecular brightness of environment-sensitive ICG, the emission intensity was measured individually, averaged, and normalized to the integration time and molecular concentration of the dyes. At 30 μM ICG, the most emissive ICG concentration in water, the fluorescent intensity in ethanol was about 12 (and 9 times) brighter than in water in the NIR-I (and -II) regions, respectively. Increasing the ICG concentration to 500 μM, the highest concentration tested, the emission signal in ethanol was approximately 140 (and 85 times) brighter than in the water in the NIR-I (and -II) regions, respectively, when excited with 730 nm light. ICG in water has concentration-dependent quenching, with emission peaking at around 30–62.5 μM, indicating this threshold results in the maximum emission intensity. However, in ethanol, higher concentrations of ICG can be used for bright emission without significant quenching. The later NIR I and NIR II wavelengths and increased emission signal should improve applications or enable new applications by improving sensitivity and depth of penetration with reduced scatter, for example, for deeper tissue imaging in medical applications.

## 4. Materials and Methods

### 4.1. Reagents 

Indocyanine green (ICG, USP, Rockville, MD, USA), which is a United States Pharmacopeia reference standard, and anhydrous ethanol (200 proof) were purchased from Sigma-Aldrich (St. Louis, MO, USA). Deionized water was dispensed from a Milli-Q ultrapure water system. All materials and solvents were used as received without further purification. ICG was diluted in water or ethanol. To prevent the ICG J-aggregation effect, we conducted the absorption and fluorescence measurements with freshly prepared solutions on the same day of analysis. Supporting Information Figure S3 presents the differences in the absorption spectra between the freshly prepared solution in water, showing monomer and H-aggregate peaks but no J-aggregate peak, and the sample prepared in water that was stored at room temperature for one week, which contained J-aggregates.

### 4.2. Absorption Measurements 

Absorption spectra were acquired on a multi-mode multiplate reader (Synergy HTX, Agilent, Savage, MD, USA) in UV-vis absorption mode. All solutions for the sample and blank measurements were performed using a flat-bottom 96-well plate or Take3 microvolume plate. Since the dynamic range of the spectrometer is limited to optical densities from 0 to 4, we acquired two different pathlengths of 2.5 mm for low concentrations and 0.5 mm for high concentrations, respectively. By the Beer-Lambert law, we multiplied the OD values of the thin pathlength by five times for correction [18].

### 4.3. Fluorescence Measurements 

Photoluminescence spectra were obtained on a spectrofluorometer (Nanolog, Horiba, NJ, USA) with an InGaAs detector (Symphony II, Horiba, NJ) cooled down to −103 °C. The 90° angle geometry between excitation and emission beams was optimal to reduce interferences such as the scattering and reflections of excitation light. All sample solutions were measured using a 2 mm × 10 mm quartz cuvette. All of the concentration dependences for ICG in deionized water and ethanol were measured at room temperature with constant experimental conditions: excitation/emission’s center wavelength/bandwidth, slit width, and data acquisition time. The Xe lamp with a monochromator centered at 730 nm with a 3 nm bandwidth was used as the excitation light. The emission light was collected in the monochromator with a near-infrared diffraction grating (100 grooves/mm blazed at 780 nm). The emission spectrum was acquired five times in 1 s each. The reported emission spectra were averaged from five spectra and subtracted from the background spectrum with a dark count of the InGaAs detector.

### 4.4. Data Analysis 

Absorption and emission spectra were analyzed in Excel (ver.2202, Microsoft Office, Redmond, WA, USA), Origin (2022b, OriginLab, Northhampton, USA), and Matlab (R2022a, MathWorks, Natick, MA, USA) programs. 2D-COS (two-dimensional correlation spectroscopy) analysis was performed using Origin and Matlab. Normalized absorption spectra were divided by the highest absorbance value. 2D correlation spectroscopy, which involves incorporating multivariate chemometrics techniques to improve the data quality for 2D correlation analysis, is one of the most powerful methods to explore correlations between perturbation-induced spectral responses [35]. Spectrally integrated fluorescent intensity was averaged in two regions of interest: 800 nm–1000 nm for NIR-I and 1000 nm–1200 nm for NIR-II, respectively. For further spectral analysis, classical least squares methods [36] were applied so that the ICG absorbance is a superposition of monomer and H-aggregate forms, and the fluorescence spectra are the combination of two bands at 820 nm and 880 nm. The spectral analysis of the fluorescence spectra was performed by nonlinear least squares curve fitting in Matlab software (version 9.12).

## 5. Conclusions

ICG forms and emissions are solvent-dependent. Monomers and H-aggregates are formed in water, whereas essentially only the monomer is found in ethanol. Emission is by the monomer form, not H-aggregates, which instead may inhibit total emission as ICG concentration increases in water. For the first time experimentally, we demonstrate two emission patterns at 820 nm and 880 nm consistent with two monomer conformations, with the second consistent with a 55° (or 125°) twist compared to the planar monomer form. This further red-shifts emission close to NIR-II. In addition, emission in ethanol does not plateau at low concentrations (~30–62 μM concentration in water) but instead increases with increasing ICG concentration to the levels tested with 730 nm excitation. The emission is ~12–140 times brighter at NIR-I and ~9–85 times brighter at NIR-II in ethanol vs. water, depending on ICG concentration. The finding may guide the creation of new fluorescent agents as well as new medical and non-medical applications.

## Data Availability

The data presented are contained within the article, and raw data available upon request.

## Supplementary Materials

The supporting information can be downloaded at: https://www.mdpi.com/article/10.3390/ijms241713030/s1.
