# Supplementary material for: Indocyanine Green (ICG) Fluorescence Is Dependent on Monomer with Planar and Twisted Structures and Inhibited by H-Aggregation
Source: Int J Mol Sci. 2023 Aug 22;24(17):13030. doi: 10.3390/ijms241713030 (PMC10488082; doi:10.3390/ijms241713030)
Supplement: Supplementary file 1 [file ijms-24-13030-s001.zip › ijms-2467047-supplementary.pdf]

Supporting Information

Indocyanine green (ICG) fluorescence is dependent on monomer with planar and twisted structures and inhibited by H-aggregation

Bonghwan Chon[1], William Ghann[2], Jamal Uddin[2], Bahman Anvari[3] and Vikas Kundra [1,4*]

[1] Department of Diagnostic Radiology and Nuclear Medicine, University of Maryland School of Medicine, 22 S. Greene St., Baltimore, MD 21201, United States

[2] Center for Nanotechnology, Department of Natural Sciences, Coppin State University, 2500 W North Ave, Baltimore, MD 21216 Baltimore, MD 21216, United States

[3] Department of Bioengineering and Department of Biochemistry, University of California, Riverside, 900 University Ave, Riverside, CA 92521 Riverside, CA 92521, United States

[4] Marlene and Stewart Greenebaum NCI Comprehensive Cancer Center Program in Oncology, Experimental Therapeutics, University of Maryland School of Medicine, 22 South Greene Street, Baltimore, MD 21201, United States

*** Correspondence: Vikas Kundra: vkundra@som.umaryland.edu**

Contents

Figure S1. Absorption spectra of ICG dissolved in water and ethanol for low concentration
Figure S2. Intensity-corrected fluorescence spectra of ICG in (a) water and (b) ethanol, respectively.
Figure S3. Representative absorption spectra of ICG solution of freshly prepared ICG solution vs. ICG solution stored at ambient temperature for one week in (a) water and (b) ethanol, respectively

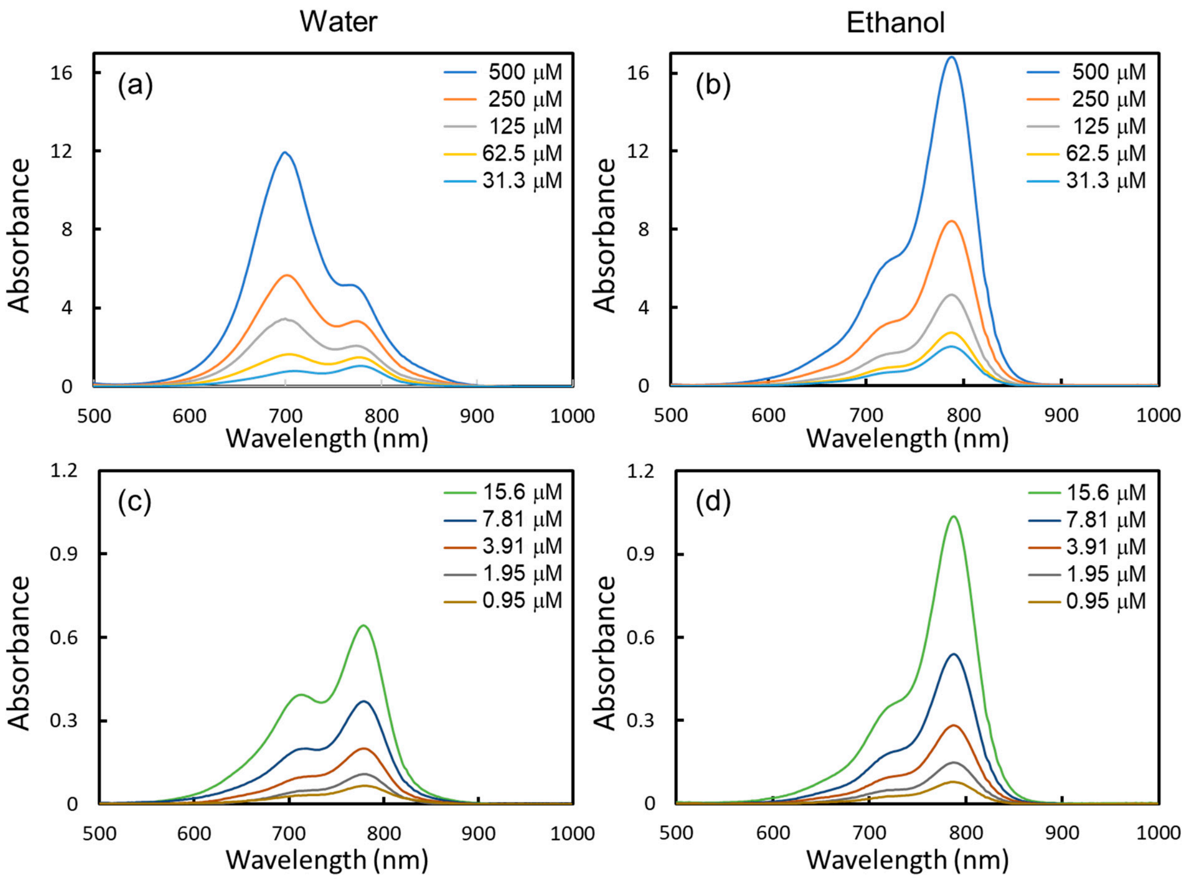

Figure S1. Absorption spectra of ICG dissolved in (a, c) water and (b, d) ethanol for high (500 – 31.3 µM) and low (15.6 – 0.95 µM) concentrations

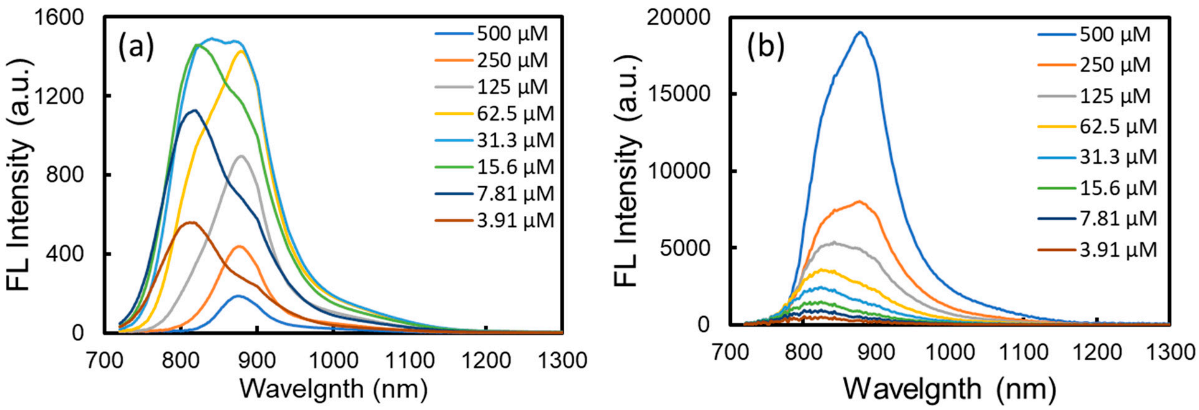

Figure S2. Intensity-corrected fluorescence spectra of ICG in (a) water and (b) ethanol, respectively. The fluorescence intensity was corrected by the sensitivity of a linear InGaAs array detector as provided by the company.

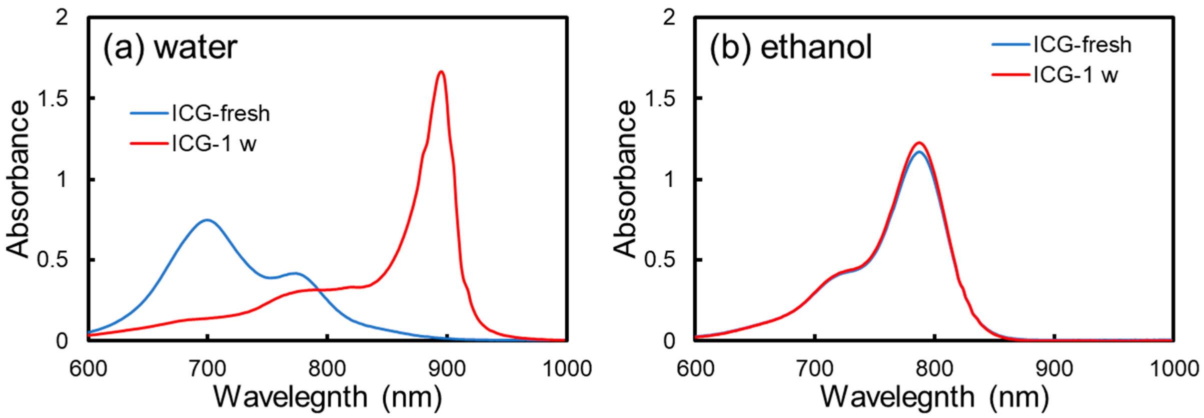

Figure S3. Representative absorption spectra of 125 μM ICG solution in (a) water and (b) ethanol for freshly prepared sample (blue) vs. another sample stored at ambient temperature for one week (red), corresponding to H-aggregates (700 nm), monomer (780 nm), and J-aggregates (890 nm). The absorption spectra of the freshly prepared sample were measured on the same day, while the sample that was stored at room temperature for one week was measured one week later after preparation.
